# The role of paracrine crosstalk between myeloid and endothelial cells in myocardial angiogenesis and infarcted heart repair

**DOI:** 10.20517/jca.2022.37

**Published:** 2023-01-01

**Authors:** Kyu-Won Cho, Seongho Bae, Young-sup Yoon

**Affiliations:** 1 Department of Medicine, Division of Cardiology, Emory University School of Medicine, Atlanta, GA 30322, USA.; 2 Severance Biomedical Science Institute, Yonsei University College of Medicine, Seoul 03722, Korea.

Ischemic heart disease is one of the leading causes of morbidity and mortality in the USA. It is mainly caused by the narrowing or occlusion of coronary arteries by plaque buildup, leading to a limited supply of oxygen and nutrients to the cardiac muscle. This results in necrotic death of cardiomyocytes (CMs). CM necrosis leads to the production of cytokines, chemokines, and damage-associated molecular patterns (DAMPs), which recruit immune cells from the bone marrow (BM)^[1]^. Infiltrated immune cells secrete proteases and cytokines that mediate inflammatory responses and fibroblast activation^[1]^. Subsequently, the damaged cardiac muscle is replaced with extracellular matrix produced by activated fibroblasts, leading to myocardial remodeling and dysfunction. Attempts to restore blood vessels (a.k.a. therapeutic angiogenesis) reduced fibrosis and improved the performance of the infarcted heart^[2]^. A possible underlying mechanism is that the supply of oxygen and nutrients via new blood vessels would preserve CM survival and support the health and function of remaining cardiovascular cells, thereby preventing adverse cardiac remodeling. Thus, therapeutic angiogenesis has been considered one of the important therapeutic approaches for ischemic heart diseases.

Investigations in the late 1990s and early 2000s indicated that the BM contains stem/progenitor cells, which can differentiate into multiple cell types. These results instilled great hope and excitement for cell therapy with BM-derived cells. However, clinical trials showed that injection of unselected bone marrow mononuclear cells (BMMNCs) into the infarcted heart only modestly improved cardiac function^[2]^. Studies raised questions regarding the mechanism of BM-derived cells-mediated cardiac repair. The observation that BM-derived cells directly transdifferentiate into CMs was refuted by other subsequent studies. Instead, it was revealed that BM-derived cells promote cardiac repair via short-term paracrine or humoral effects^[3]^. Over the past decade, genetic lineage tracing and single-cell RNA-seq approaches helped us better understand the differential roles of subpopulations in BMMNCs during cardiac remodeling and repair. Among them, myeloid cells play important roles during the sterile inflammation of the heart. They infiltrate into the damaged heart, engulf cell debris, and secrete a variety of paracrine factors to communicate with other cell types^[1]^. Emerging evidence suggests that myeloid cells promote vascular repair by secreting paracrine factors. Thus, identification and functional characterization of myeloid cell-derived paracrine factors can be critical to better understanding the pathophysiology of cardiovascular diseases.

Reboll and colleagues identified paracrine crosstalk between myeloid and endothelial cells (ECs) for vascular repair during cardiac ischemic injury responses^[4]^ [Figure 1]. They found that Meteorin-like (METRNL) secreted by infiltrated myeloid cells binds to KIT tyrosine kinase receptor expressed on a subset of ECs, promoting proliferation and migration of KIT^+^ ECs. METRNL was known to be produced by inflammatory macrophages, but its receptor had been unknown. By utilizing genetically engineered mice and cutting-edge techniques, Reboll *et al*. demonstrated that METRNL-KIT signaling is crucial for vascular regeneration and cardiac repair. Stem cell factor (SCF) is a previously known ligand for KIT^[4]^. Reboll *et al*. showed that both METRNL and SCF bind to KIT with high affinity^[4]^. In contrast to METRNL, however, SCF was not upregulated in the infarcted heart. Moreover, major source cells for SCF in the heart are ECs, fibroblasts, and CMs. This suggests that there is minimal competition between METRNL and SCF for binding to KIT in the injured heart. If so, we can ask what the function of METRNL in a steady state without injury would be. While mice lacking *Scf* or *Kit* are embryonically lethal, *Metrnl* knockout mice were viable without apparent defects. There are two possibilities for the divergent observations. First, the prenatal expression level of METRNL might be negligible. Second, SCF might play compensatory roles for METRNL signaling in the METRNL knockout mice. It would be interesting to examine the prenatal expression of METRNL, its molecular interplay with SCF, and its impact on embryogenesis and organogenesis.

In addition, Reboll *et al*. made an interesting observation on the role of infiltrated macrophages (CCR2^high^) versus resident macrophages (CCR2^low^) in vascular repair^[4]^. Some reports claimed that BM-derived macrophages are angiogenic and reparative: infiltrated macrophages expressed higher angiogenic factors such as VEGF and MYDGF, whereas resident macrophages expressed pro-inflammatory factors such as TNFα and TGFβ1^[5,6]^. However, others reported that adult BM-derived macrophages are not helpful for angiogenesis and cardiac regeneration: inhibition of macrophage recruitment by a CCR2 inhibitor increased vascular density after cardiac injury^[7]^. In addition, resident macrophages that arise from the yolk sac were shown to promote angiogenesis^[7]^ and prevent adverse cardiac remodeling^[8]^. In this study, Reboll *et al*. showed that METRNL is primarily expressed in infiltrated macrophages (CCR2^high^) rather than resident macrophages (CCR2^low^), claiming that BM-derived macrophages are essential for vascular repair^[4]^. Further studies are needed to address this controversy of whether angiogenic myeloid cells may exist in subpopulations of both recruited and resident macrophages.

Furthermore, Reboll *et al*. add a new perspective to the endothelial heterogeneity in the heart^[4]^. They showed that there is a subset of cardiac ECs that express KIT and they proliferate in METRNL- or KIT-dependent manners under ischemic stress. On the other hand, KIT-negative ECs were expanded independently of METRNL-KIT signaling, suggesting that different subsets of ECs proliferate via distinct mechanisms in response to myocardial infarction. However, it is still unclear how only a subset of ECs express KIT but others do not. First, it is possible that KIT^+^ ECs have different developmental origins from other ECs. ECs can originate from angioblasts (mesoderm-derived endothelial precursors) and erythromyeloid progenitors. Thus, the origin of Kit^+^ ECs versus Kit^−^ ECs remains to be determined. Using genetic lineage tracing approaches, van Berlo *et al*. showed that c-kit^+^ cells generate ECs in the heart in both physiological and pathological conditions^[9]^. The proportion of c-kit lineage ECs doubled after myocardial infarction^[9]^, consistently with Reboll *et al*., where the population of Kit^+^ ECs increased in the infarcted heart^[4]^. Since c-kit is expressed in a variety of cell types, dissecting the origin of Kit^+^ ECs and manipulation of the precursor cells to promote the generation of KIT^+^ ECs could be an intriguing research subject for vascular repair and its resultant heart regeneration. Second, it is possible that KIT expression could be induced on ECs. A study showed that KIT expression was induced in mature human CD8^+^ T cells after primary activation^[10]^. It would be worthwhile to define the pathophysiologic conditions and molecular mechanisms that induce endothelial expression of KIT. Understanding the regulation of KIT expression on ECs could maximize the therapeutic potential of METRNL for vascular repair.

In summary, Reboll *et al*. revealed that a novel form of molecular crosstalk between infiltrated myeloid cells and a subset of cardiac endotshelial cells drives angiogenesis and tissue repair after myocardial infarction^[4]^. Furthermore, the authors identified METRNL as a key myeloid paracrine factor that induces the proliferation of KIT^+^ ECs. This study offers new perspectives on the controversy about the role of infiltrated macrophages in vascular regeneration and on the heterogeneity of cardiac ECs. Furthermore, this study lays the foundation for new research topics in the fields of cardioimmunology and vascular biology. Addressing the remaining questions will help us to better understand molecular and cellular mechanisms underlying ischemic heart disease and to develop innovative therapeutic strategies.

## Figures and Tables

**Figure 1.**
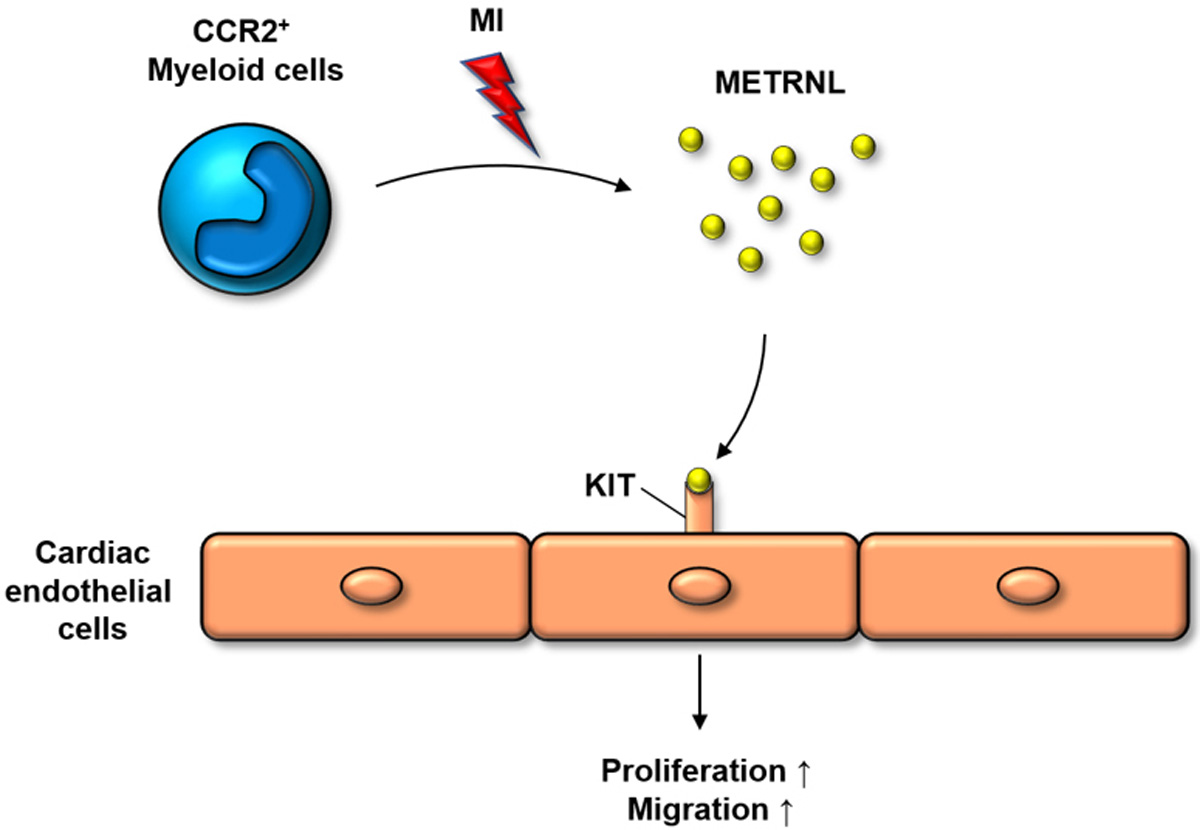
A schematic summary for the paracrine crosstalk between myeloid and endothelial cells (ECs) for vascular repair of the injured heart. In response to myocardial infarction (MI), CCR2^+^ myeloid cells infiltrate the heart and secrete METRNL which binds to KIT tyrosine receptor expressed on a subset of cardiac ECs. The KIT-METRNL signaling cascade induces the proliferation and migration of KIT^+^ ECs, contributing to tissue repair.

## Data Availability

Not applicable.
